# From soils to lake: interplay between hydrology and local environmental settings drives species selection across a karst landscape

**DOI:** 10.3389/fmicb.2026.1813326

**Published:** 2026-05-13

**Authors:** Anusha Priya Singh, Paul-Adrian Bulzu, Vojtech Lanta, Pavel Chaloupský, Michaela M. Salcher, Tanja Shabarova

**Affiliations:** 1 Department of Aquatic Microbial Ecology, Institute of Hydrobiology, Biology Centre CAS, Ceske Budejovice, Czechia; 2 Faculty of Science, University of South Bohemia, Ceske Budejovice, Czechia; 3 Department of Functional Ecology, Institute of Botany CAS, Pruhonice, Czechia; 4 Department of Chemistry and Biochemistry, Faculty of AgriSciences, Mendel University in Brno, Brno, Czechia

**Keywords:** core microbiome, karst, microbial community composition and diversity, network analysis, terrestrial-aquatic interface

## Abstract

Terrestrial and aquatic ecosystems are interconnected through runoff and hydrological networks that facilitate the transfer of microbial communities across landscapes. While microbial transport along surface waters is well documented, the role of subsurface hydrological paths in shaping microbial community composition remains poorly understood, particularly in complex karst systems. Here, we studied bacterial communities under stable hydrological conditions across a peri-alpine karst landscape, where mixed limestone-sandstone catchments drain via both surface and subsurface hydrological networks into Lake Thun (Switzerland). We profiled 16S rRNA gene sequences from soils, sediments, surface and subsurface waters, and distinct lake strata. All environments except the lake exhibited high microbial diversity. We observed a clear transitional gradient in bacterial communities along the terrestrial-aquatic interface, with environment type explaining 19% of total variation. Core microbiome analyses revealed both environment-specific and shared taxa, with the strongest overlap between surface and subsurface hydrological networks (63.8%–84.6% shared core taxa). Co-occurrence network analysis identified six major modules. Three of them represented distinct metabolic assemblages tightly associated with specific environment types: peat soils, lake strata, and the subsurface network, respectively. One recurrent module spanned multiple environments and was linked to redox-driven processes, including the oxidation of nitrogen compounds, metals, and methane. Two additional modules comprised aquatic copiotrophs associated with streams and soil heterotrophs prone to export and short-term persistence within the hydrological network. Overall, our results demonstrate that specific environmental settings and hydrological connectivity jointly contribute to selection of microbial species within the karst landscape.

## Introduction

Runoff and hydrological networks often facilitate the transfer of biotic and abiotic components between terrestrial and aquatic environments and shape the composition and distribution of microbial communities across multiple scales and interfaces (Gounand et al., 2018; Stadler and del Giorgio, 2022) including soils and headwater streams (Caillon et al., 2021; Ruiz-González et al., 2015; Niño-García et al., 2016, 2017), or sediment and the water column (Zhang et al., 2021). This influence is also detectable along the stream-river continuum (Besemer et al., 2012; Crump et al., 2007, 2012; Savio et al., 2015). However, little is known about microbial community transfers and compartmentalisation within interconnected surface and subsurface environments (Zhu et al., 2021; Ruff et al., 2024).

Terrestrial and aquatic microbiomes are influenced by local physico-chemical parameters. Soil microbes respond to pH, moisture content, vegetation cover, type of parent bedrock, and temperature (Lauber et al., 2009; Griffiths et al., 2011; Terrat et al., 2017). Aside from pH (Ruiz-González et al., 2015) and water temperature (Hullar et al., 2006), aquatic microbial communities are also influenced by dissolved oxygen (Fusi et al., 2023) and nutrient concentrations (Newton et al., 2011; Logue et al., 2012), water retention time (Ruiz-González et al., 2015; Niño-García et al., 2016), as well as streambed characteristics (Mendoza-Lera et al., 2017).

The downstream transport of dissolved organic matter and microbial taxa from terrestrial sources into aquatic environments is well known (Crump et al., 2007, 2012; Besemer et al., 2012). Conceptual frameworks such as the soil-headwaters continuum propose that microbial transport within terrestrial catchments proceed laterally through soils before entering aquatic networks (Fierer et al., 2007). Microbial community simplification in aquatic systems usually follows a downstream gradient and is reflected by declining alpha diversity (Besemer et al., 2012; Ruiz-González et al., 2015). However, in landscapes where streams divide into parallel surface and subsurface waterways, this process is expected to become increasingly complex and less predictable. How microbial communities are distributed across these parallel pathways, and the extent to which they exchange between surface and subsurface domains, remains poorly understood.

Studies of aquatic karst subsurface components, including springs, cave pools, and cave streams, indicate the presence of a locally persistent core microbiome predominant in these environments under low-flow conditions (Farnleitner et al., 2005; Pronk et al., 2006; Shabarova et al., 2014; Savio et al., 2019; Pedron et al., 2022). This persistence is usually linked to long water residence times and interrupted hydrological connections, which promote strong environmental filtering and taxa adapted to stable subsurface conditions. At the same time, these environments show high sensitivity to hydrological disturbance. Recharge events and increased flow rapidly alter microbial assemblages through surface-derived inputs. Such events are followed by successional recovery toward low-flow community states once hydrological stability is restored (Pronk et al., 2006; Shabarova et al., 2014; Brannen-Donnelly and Engel, 2015; Savio et al., 2019). These observations identify hydrological regime and water residence time as primary drivers of microbial community composition in karst subsurface. In addition to hydrology, catchment elevation, water chemistry, and seasonality have been shown to influence bacterial communities in subsurface aquatic environments (Farnleitner et al., 2005; Shabarova and Pernthaler, 2010; Shabarova et al., 2014; Slabe et al., 2021).

While hydrological connectivity is recognized as a driver of microbial community assembly, studies integrating terrestrial environments with both surface and subsurface aquatic networks across landscapes remain rare. Here, using 16S rRNA gene amplicon sequencing, we studied bacterial community composition across a peri-alpine karst landscape on the northern shore of Lake Thun (Switzerland), encompassing soils from diverse parent-rock and vegetation types, sediments, surface waters, subsurface aquatic components, rivers, and two lake strata, sampled under hydrologically stable conditions. By focusing on periods of low and stable flow, we aimed to access baseline community patterns under minimal active dispersal and mixing. We hypothesized that under such conditions, microbial community structure would reflect environmental filtering and hydrological compartmentalization, resulting in distinct but partially connected microbial assemblages across terrestrial, and surface and subsurface aquatic systems. Consistent with this hypothesis, our results reveal varying degrees of connectivity among environments, both across and within terrestrial and aquatic boundaries, and identify environment type-specific, niche-based as well as transfer driven microbial signatures across the karst landscape.

## Materials and methods

### Site description

The sampling site (Supplementary Figure S1), located on the northern shore of Lake Thun (Switzerland), includes both surface and karst catchments dominated by subsurface discharge through an extensive underground drainage network. Both surface and subsurface aquatic systems ultimately drain into Lake Thun. This lake is a peri-alpine oligotrophic system situated on the northern edge of the Alps (46.6958° N, 7.7212° E), with a maximum depth of 216.5 m (Cojean et al., 2021). The karst areas at the study site host some of the most extensive cave systems in the world (Häuselmann, 2002), with a combined explored length of approximately 320 km, ranging in elevation from 480 to 2000 m a.s.l (Jeannin and Häuselmann, 2012; Häuselmann, 2018). Two main catchments are recognized: the smaller is represented by the St. Beatus Caves system (12 km), and a larger one extends from the Bätterich and Gelbenbrunnen springs at Lake Thun through caves such as Bärenschacht, Faustloch, F1, K2, and others to the Siebenhengste-Hohgant cave system (Häuselmann, 2002, 2018). The surface flow is represented by the Lombach stream (the fourth largest inflow to Lake Thun), along with permanent streams such as the Grönbach and temporary ones such as the Sundbach (Jeannin and Häuselmann, 2012). The region features a patchy geological composition, dominated by sedimentary rocks, primarily limestone (LS), quartz-sandstone (QS), their mixtures (calcareous sandstone, CS), along with flysch (FL), and less-defined debris (schutt, ST) in the stream beds and on slopes (Luetscher et al., 2021). The landscape exhibits a diverse mosaic of vegetation zones, including grasslands, forests (spruce and mixed), bogs (encompassing wetlands, flat bogs, and raised peat bogs), and areas with limestone pavements (see Supplementary Text for details).

### Sampling

The main sampling campaign was conducted in September 2020 during stable hydrological conditions (n samples = 132, Supplementary Table S1) and included both surface and subsurface components of the hydrological network (pools, streams) as well as the two main rivers that drain into Lake Thun (Aare and Kander), along with different lake strata (epi- and hypolimnion) and soil samples encompassing distinct vegetation types and bedrock formations (mentioned above). Previous (2018, 2019) and subsequent (2021, 2022) sampling campaigns conducted under comparable hydrological and seasonal conditions provided additional samples along central hydrological flow paths, including surface and subsurface waters, lake environments, soils and sediments (*n* = 69). Additionally, samples from European karst and non-karst lakes (*n* = 19) (Salcher et al., 2025) were included to increase the size of the lake dataset and improve the robustness of within-group analyses of alpha diversity.

Water samples from the surface streams (*n* = 58), pools (*n* = 6), rivers (*n* = 4), and lake (*n* = 10) were collected in sterile bottles. Water was pre-filtered using 20 μm mesh and filtered on site through Sterivex polyethersulphone filter units (0.22 μm pore size, Millipore, USA) using a peristaltic pump until the filter was saturated with biomass. The filter units were heat sealed on both ends after adding 0.75 mL DNA–RNA shield (Zymo Research, USA), transported to the lab and stored at −80 °C until further processing. Eight water samples from Lake Thun’s deepest point were collected with a Niskin bottle from two depths (5 and 180 m) during four sampling campaigns. Two additional samples were taken next to the underwater spring Bätterich at 1 and 6.5 m depths. Water samples from subsurface pools (*n* = 25) and streams (*n* = 22) collected in the caves were taken with 50 mL syringes and manually filtered through Sterivex filters. A complete list of all aquatic samples is presented in the Supplementary Table S1.

Soil samples (*n* = 53) were collected from the following parental rock environments: quartz-sandstone, calcareous sandstone, limestone, flysch and schutt; encompassing several vegetation types, including forest and grassland (F + GL), limestone pavement (LP), and bog (PB). Sampling sites included locations situated within the lateral expansion zone of streams as well as sites without stream contact; stream presence or absence at each location was recorded. At each site, a composite sample of five soil cores - four from the corners and one from the center - of a randomly selected 5 m × 5 m plot were taken from the A-horizon (depth varying from 5 to 40 cm). The collected fractions were pooled, sieved through a 5 mm mesh to remove plant debris and small stones and a homogenized subsample was transferred to a sterile 50 mL polypropylene vial and stored at 4 °C until further processing. In the laboratory, duplicates of 200 mg of soil were aliquoted into sterile 2 mL microcentrifuge tubes, amended with 500 μL of DNA/RNA Shield and stored at −80 °C until DNA extraction. Water content of soil samples was determined gravimetrically at 65 °C until a constant weight was achieved (modified from Dettmann et al., 2021). Soil pH was measured by mixing 10 g of soil with Milli-Q water at a 1:5 (w/v) ratio. The suspension was shaken for several minutes, allowed to settle, and the pH was measured using a YSI pH probe (Faria et al., 2023). Composite samples representing the upper layer of the stream sediment were collected at five points across the stream bed, using a plastic tube for surface (*n* = 8) and cave streams (*n* = 13). Pooled samples were frozen within 24 h and stored at −20 °C until delivered to the lab where they were processed with same protocol as the soil samples. The complete metadata of soil and sediment samples is present in the Supplementary Table S2.

### Water chemistry

Water filtered through Sterivex filters was collected in plastic amber bottles (250 mL) for the analysis of physico-chemical parameters as described before (Park et al., 2023). This included ion chromatography for cations (Mg^2+^, Na^+^, K^+^, Ca^2+^, NH_4_^+^) and anions (Cl^−^, SO_4_^2−^, F^−^, NO_2_^−^, NO_3_^−^), pH, conductivity and alkalinity and concentrations of dissolved reactive phosphorus (DRP), dissolved phosphorus (DP), dissolved organic carbon (DOC), and dissolved nitrogen (DN). Additionally, vertical profiles for pH, temperature, conductivity, dissolved oxygen, and chlorophyll *a* concentration were taken during lake sampling using a submersible multiparametric probe (YSI EXO3, Yellow Springs Instruments, Yellow Springs, OH, USA). All chemistry measurements for water samples are provided in Supplementary Table S1.

### DNA extraction and amplification of 16S rRNA gene fragments

Genomic DNA was extracted using the Quick-DNA Faecal/Soil Microbe Kit (D6012, Zymo Research, USA) with slight modifications. Briefly, the Sterivex filter units were opened, the filters were cut on sterile Petri dishes and added to BashingBead™ Lysis Tube along with the DNA–RNA shield. The soil and sediment samples were directly transferred to BashingBead™ Lysis Tube. Bead beating was executed on a vortex adapter fitted with tube holder assembly (Vortex-Genie 2, Mo Bio Labs, USA) for 15 min at maximum speed. The collected filtrate was amended with a Genomic Lysis buffer (400 μL of 95% ethanol was added to soil and sediment samples). DNA extraction was done according to the kit manufacturer’s instructions, and concentrations were quantified using a Qubit 3 Fluorometer using the Qubit dsDNA HS Assay kit (Q32850, Thermo-Fischer Scientific, USA). Primers 515f and 926r (Parada et al., 2016), flanked by CS1 and CS2 adapters (Fluidigm Corporation, USA) were used to amplify the V4-V5 region of 16S rRNA genes. PCR was carried out using Q5 High Fidelity Mastermix (New England Biolabs, USA) under the following conditions: initial denaturation at 98 °C for 30 s, followed by 28 cycles of denaturation (98 °C/15 s), annealing (50 °C/60 s) and extension (72 °C/90 s); final extension step lasted for 7 min at 72 °C.

### Library preparation, amplicon sequencing and sequence analyses

Sequencing libraries were prepared from PCR products using unique labelling (Fluidigm Corporation, USA) and equimolar aliquots were sequenced on an Illumina MiSeq platform at Genomics and Microbiome Core Facility of Rush University (V2 chemistry for 2 × 250 paired reads), with an average sequencing depth of 15,000 raw reads per sample. Cutadapt version 4.7 (Martin, 2011) was used for removing primer sequences. The trimmed sequences were merged and dereplicated using R package dada2 version 1.28 (R version 4.3.0) (Callahan et al., 2016) with default parameters and reads’ trimming adjusted according to the observed quality. Forward reads were truncated at 230 bp, reverse reads at 200 bp and reads with expected error rates exceeding 3 were removed. Additionally, chimeric sequences were removed using UNOISE3 (minsize = 8) in USEARCH11 (Edgar, 2010, 2016). Taxonomic assignment was done using SILVA database v138.1 (https://www.arb-silva.de/, Quast et al., 2013). The obtained taxonomic assignments were mapped to the Genome Taxonomy Database (GTDB) nomenclature where possible (Parks et al., 2022). To validate sequencing accuracy and assess potential contamination, 12 blank DNA extractions and nine mock communities were included in the sequencing runs alongside the environmental samples (ZymoBIOMICS® Microbial Community DNA Standard and Standard II, ZymoResearch, USA). Mock communities served as reference standards to evaluate efficiency of chimera removal and to detect contaminations (Karstens et al., 2019). Amplicon sequence variants (ASVs) were ranked by relative abundance within each mock community and compared to the expected composition as provided by the manufacturer’s reference datasheet. To ensure data quality, samples with reads lower than 15,000, post merging of replicates, were excluded from the analysis, and then data set was normalized to the same value using the rarefy function in R package vegan version 2.6–8 (Oksanen et al., 2018).

### Statistical analysis

Statistical analysis was conducted using the R environment version 4.2.3 (R Core Team, 2020). Alpha diversity metrics (observed richness and Shannon’s index) among the different environment types were calculated using R package vegan and compared using the Kruskal–Wallis test along with Dunn’s *post-hoc* pairwise comparisons (with Benjamini-Hochberg adjustments) using R package FSA version 0.10.1 (Ogle et al., 2026). Nonmetric multidimensional scaling (NMDS) based on Bray-Curtis dissimilarities was used to visualize distances between bacterial communities across the landscape using R packages phyloseq version 1.40.0 (McMurdie and Holmes, 2013) and vegan. Differences in microbial communities was evaluated by permutational multivariate analysis of variance (PERMANOVA) using the adonis2 function. Pairwise comparisons between sample types were performed using the pairwiseAdonis R package version 0.4.1 (Arbizu, 2020). Multivariate homogeneity of group dispersions was tested using PERMDISP (betadisper function) to evaluate whether significant PERMANOVA results were due to differences in group centroids or dispersions. An additional NMDS was performed on phylum-level data. Further multivariate analyses were conducted in CANOCO version 5.1 (ter Braak and Smilauer, 2012) to assess the influence of physicochemical parameters on phyla distribution in soil and aquatic environments. Samples from the 2020 campaign supplemented by four epilimnion and hypolimnion samples from summer and autumn campaigns in 2018–2019 were used for this purpose. Environment type was included as a supplementary variable, while geographic coordinates and altitude were used as covariates for soil samples only. Gradient length was evaluated to guide method selection. Identification of core taxa was done using a prevalence-abundance filtering approach adapted from R package Microbiome (Lahiti and Shetty, 2017). Within each environment, two metrics were calculated: (i) prevalence, i.e., the proportion of samples in which an ASV was present, and (ii) mean relative abundance across samples. A core microbiome subset of ASVs present in ≥50% of samples with a mean relative abundance ≥0.1% were prepared (Neu, 2021). A different filtering approach (relative abundance ≥0.5% and prevalence in ≥4 samples) was used for the whole karst dataset. For both subsets, co-occurrence network analysis was performed using modified R scripts available at https://github.com/RichieJu520/Co-occurrence_Network_Analysis (Hu et al., 2017) using Spearman’s correlation coefficient (|*ρ*| > 0.6) and significance level <0.01. Networks were visualised using Gephi version 0.10.1 (Bastian et al., 2009). Functional profiles for the network members across the whole karst dataset were predicted with the FAPROTAX database using R package microeco (1.2.4) (Liu et al., 2021) and summarized for every metabolic module. Additionally, ggplot2 (version 3.5.2; Wickham, 2016) and ComplexHeatmap (Gu et al., 2016) were used for data visualization.

## Results

### Overview of data processing and quality control

We sequenced a total of 403 samples from soil (*n* = 164), sediment (*n* = 50), water (*n* = 164), and controls (*n* = 25) (including duplicates, Supplementary Figure S1, Supplementary Tables S1, S2). Following dada2 processing and removal of additional chimeric sequences with UNOISE3, 66,678 ASVs (92.3% of total ASVs) were retained. As Bray–Curtis dissimilarity analysis (Supplementary Table S3) revealed small distances between duplicates, confirming high reproducibility, replicates were merged for further analysis. A total of 280 ASVs (read abundance >10) were identified as contaminants from mock communities and negative controls and hence removed from the dataset (Supplementary Table S4). Samples with low reads were also removed, resulting in a final dataset of 208 samples containing 63,460 ASVs (soil: *n* = 53; sediment: *n* = 21; water: *n* = 142).

### Alpha diversity variation across environment types

Alpha diversity was significantly higher in soils and streams (Shannon Index > 6.00, observed richness > 862.72) than in all other habitats (Shannon Index = 4.04–6.00, observed richness = 223.2–843.93; Supplementary Figure S2). Quartz-sandstone (QS) soils had comparatively lower Shannon and observed diversity than other soil types [calcareous sandstone (CS), limestone (LS), schutt (ST) and flysch (FL)]. Pools and lake samples showed the lowest diversity values. Kruskal–Wallis tests indicated highly significant differences in alpha diversity among the sampled environments for both Shannon (*χ*^2^ = 103.73, *p* = 3.136 × 10^−16^) and observed richness (*χ*^2^ = 102.86, *p* = 4.628 × 10^−16^). Pairwise comparisons confirmed that community structure and diversity differed significantly between soil and lake environments, whereas subsurface and surface sediments, pools and streams exhibited intermediate characteristics, showing no significant distinction from both systems (Supplementary Figures S2A,S2B). Patterns in observed richness generally mirrored those of Shannon diversity (*r* = 0.84, *p* < 0.001).

### Community transitions within the karst landscape

**Figure 1:**
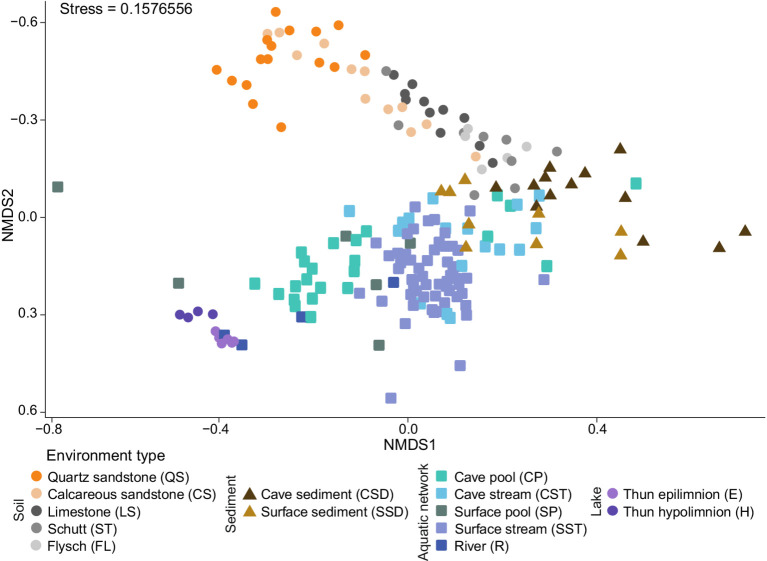
Nonmetric multidimensional scaling (NMDS) plot based on Bray-Curtis dissimilarities between prokaryotic communities resolved at ASV level. Colors and shapes indicate environment type.

**Figure 2:**
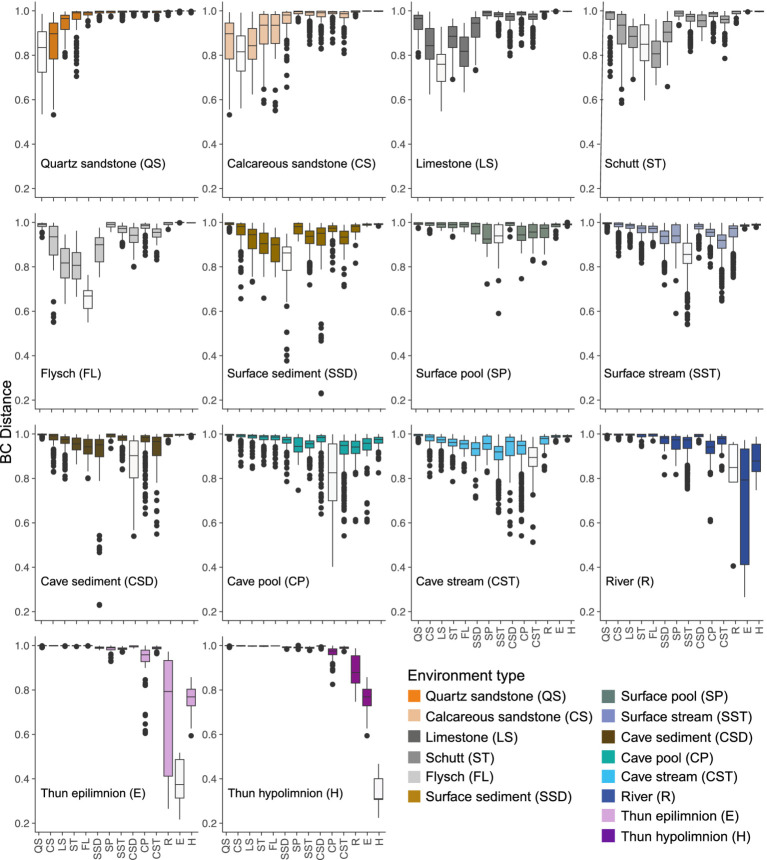
Boxplots representing pairwise Bray-Curtis dissimilarities within and among microbial communities across different environment types. Each panel represents an environment type. White boxes represent the distances within the indicated environment.

Nonmetric multidimensional scaling (NMDS) of ASVs based on Bray–Curtis dissimilarity revealed a clear transition of bacterial communities along the terrestrial-aquatic interface, characterized by gradual compositional shifts throughout the environment types, except for a clearly defined lake cluster that included several river samples (Figure 1). Environment type explained 19% of the total variation in the bacterial community composition (PERMANOVA; *F* = 3.19, R^2^ = 0.19, *p* = 0.001). Despite heterogeneous dispersions (PERMDISP; *F* = 54.41, *p* = 0.001), pairwise PERMANOVA comparisons showed significant community differences among most studied environment types, especially between lake epilimnion and soil systems, though some pairs (e.g., surface/subsurface pools vs. river and lake epilimnion/hypolimnion) showed no significant separation (Figure 2; Supplementary Table S5) and closer distances as remaining environments.

**Figure 3:**
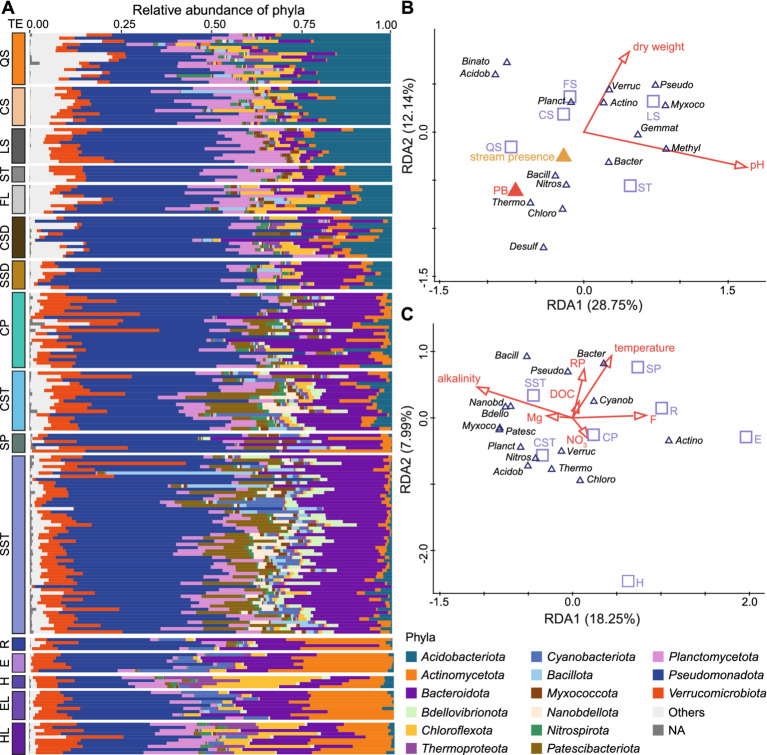
Phylum distribution within karst landscape. **(A)** Relative abundance of 15 top dominant prokaryotic phyla across environment types. Site abbreviations are defined in Figure 1, additionally, EL, HL represent epilimnia and hypolimnia of additional lakes. **(B)** Partial-RDA ordination of phyla in terrestrial environments (with altitude, latitude, and longitude as co-variates). Arrows represent numerical variables. Triangles indicate categorical environmental variables: PB - peat bog vegetation type, and presence or absence of stream. Red color indicates significant variables (*p* < 0.05), orange denotes marginal significance of *p* = 0.052. **(C)** RDA ordination of phyla in aquatic environments, constrained by environmental variables. Red arrows indicate significant forward-selected environmental variables. In both RDA ordinations, environmental types included as supplementary variables are shown as empty squares. Phylum names are abbreviated to the first six letters; *Methylomirabilota*, *Desulfobacterota*, *Gemmatimonadota,* and *Binatota* are shown only in panel **(B)**.

A phylum-level NMDS based on the distribution of 72 phyla (including 7 Archaea) reproduced the ASV-level community patterns (stress = 0.145; Mantel *r* = 0.73, *p* = 0.001; Supplementary Figure S3), indicating that general phylum-level structure captures the environmental differentiation observed at higher taxonomic resolution (Figure 3). *Pseudomonadota* (35.9%), *Planctomycetota* (7.9%), and *Verrucomicrobiota* (6.5%) occurred across all environment types, whereas the relative contributions of other phyla varied markedly.

QS soils represented the most distinct terrestrial environment, showing overlap only with CS (Figures 1, 2). *Acidobacteriota* (28.6% ± 10.4%) dominated these soils and were primarily represented by *Terriglobales* and Subgroup-2 (SILVA, release 138.1) (Figure 3A). A further characteristic of QS soils was the enrichment of *Desulfobacterota* (5.19% ± 4.17%), *Nitrospirota* (1.53% ± 2.14%), and *Thermoproteota* (1.87% ± 2.09%), whose occurrence was additionally associated with bog vegetation and stream presence, as revealed by RDA analysis (Figure 3B). *Acidobacteriota* showed a strong negative correlation with pH (Figure 3B) and gradually declined along the soil gradient CS → LS → ST → FL, decreasing from 26.1% ± 6.7% in CS to 12.8% ± 5.9% in ST. This decline was accompanied by a taxonomic shift from *Terriglobales* (15.1% ± 8.7% in QS to 0.2% ± 0.2% in FL) to *Vicinamibacterales* (1.8% ± 2.5% in CS and 8.7% ± 2.1% in FL). A similar negative relationship with pH was also observed for the *Binatota* phylum. In contrast, *Gemmatimonadota* and *Methylomirabilota* were associated with higher pH values, whereas *Verrucomicrobiota*, *Actinomycetota*, and *Myxococcales* were more prevalent in soils with lower moisture content. Altogether, pH, moisture content, vegetation type, and stream presence accounted for 42.76% of the total phylum-level variation (adjusted explained variation = 37.04%) based on forward-selected RDA models (Figure 3B).

Sediments occupied transitional positions between terrestrial and aquatic environments; however, selected cave sediment communities were clearly separated in the NMDS plot (Figure 1). Aquatic environments were characterized by higher relative abundances of *Bacteroidota* (water = 19.2 ± 7.5% vs. soil = 5.4 ± 4.4%) and *Actinomycetota* (water = 4.7 ± 7.1% vs. soil = 2.1 ± 1.7%), the latter emerging as the dominant phylum in rivers and Lake Thun (19.9 ± 11.6% in rivers, 23.3 ± 3.2% in the epilimnion, and 15.3 ± 5.6% in the hypolimnion; Figure 3C). Surface waters from rivers and Lake Thun epilimnion showed the highest relative abundances of *Cyanobacteriota* (3.9 ± 3.4% and 9.1 ± 5.6%, respectively). These taxa, together with *Bacteroidota*, *Pseudomonadota*, and *Bacillota*, were positively associated with increased temperature and elevated phosphorus and DOC concentrations (Figure 3C). *Patescibacteriota*, *Nanobdellota*, *Myxococcota*, and *Nitrospirota* reached their highest abundances in surface and subsurface streams and were associated with alkaline conditions (up to 18.4, 10.9, 6.9, and 4.7%, respectively). *Nitrospirota* were additionally more abundant in lake hypolimnion samples and cave streams (Figure 3C). *Verrucomicrobiota* showed slight enrichment in cave pools (10.1 ± 7.5%) and streams (9.2 ± 5.3%), whereas *Chloroflexota* and *Thermoproteota* were more prevalent in lake hypolimnia (10.5 ± 4.5% and 4.7 ± 5.2%, respectively). Taken together, the selected physicochemical variables explained 32.1% of the total phylum-level variation in aquatic environments (adjusted explained variation = 25.85%) based on forward-selected RDA models (Figure 3C).

### Core microbiome distribution and network analysis revealed high connectivity between the environments

**Figure 4:**
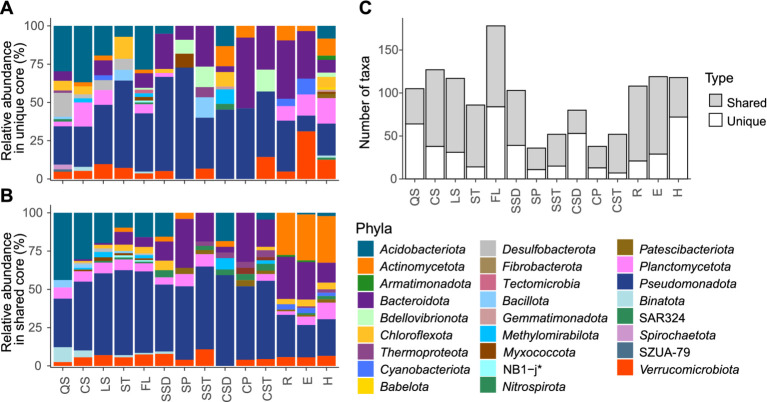
Distribution of unique and shared core taxa across environments. **(A)** Relative abundance of phyla within the unique core microbiomes. **(B)** Relative abundance of phyla within the shared core microbiomes. **(C)** Number of unique versus shared taxa detected per environment type. Phyla names with asterisks correspond to SILVA 138.1 taxonomy.

The core microbiome (defined as ASVs present in ≥50% of samples with a mean relative abundance ≥0.1%) comprised both environment-specific ASVs (unique core) and ASVs shared among two or more environments (shared core) (Figures 4A,B). Cave sediments exhibited the lowest shared core fraction (33.8%), restricted to subsurface environments. QS soils and the lake hypolimnion, located at opposite ends of the terrestrial-aquatic transition path (Figure 1), also showed limited shared cores (~39%), overlapping mainly with other soils and the lake epilimnion, respectively. In contrast, LS, ST, and FL soils showed extensive overlap not only with other soils, but also with sediment and aquatic environments. Surface sediments displayed broad connectivity, sharing 62.1% of their core ASVs. The highest levels of overlap were observed among surface and subsurface aquatic networks, which shared between 63.8 and 86.5% of their core ASVs. At the same time, cave and surface pools contained the smallest absolute numbers of core ASVs (38 and 36, respectively; Figure 4C).

Although *Acidobacteriota* (soils), *Actinomycetota* (rivers and lake strata), and *Bacteroidota* and *Pseudomonadota* dominated both core types across environments, the taxonomic composition remained site-specific even at phylum level. *Fibrobacterota* were detected exclusively in the shared cores of subsurface pools and streams and in the unique core of the lake hypolimnion. The unique core exhibited greater taxonomic diversification and was frequently dominated by low-abundance phyla, including SZUA-79 and *Spirochaetota* (exclusive to QS), *Tectomicrobia* (exclusive to FL), *Gemmatimonadota* (exclusive to cave sediments and hypolimnion), *Armatimonadota* (exclusive to epilimnion and hypolimnion), and *Babelota* and SAR324 clade (exclusive to the hypolimnion). Multiple ASVs affiliated with *Desulfobacterota* and *Bdellovibrionota* were present in the unique core; *Desulfobacterota* occurred in QS, CS, LS, and ST soils, while *Bdellovibrionota* were found in FL soils, cave sediments, surface pools and sediments, and hypolimnion.

**Figure 5:**
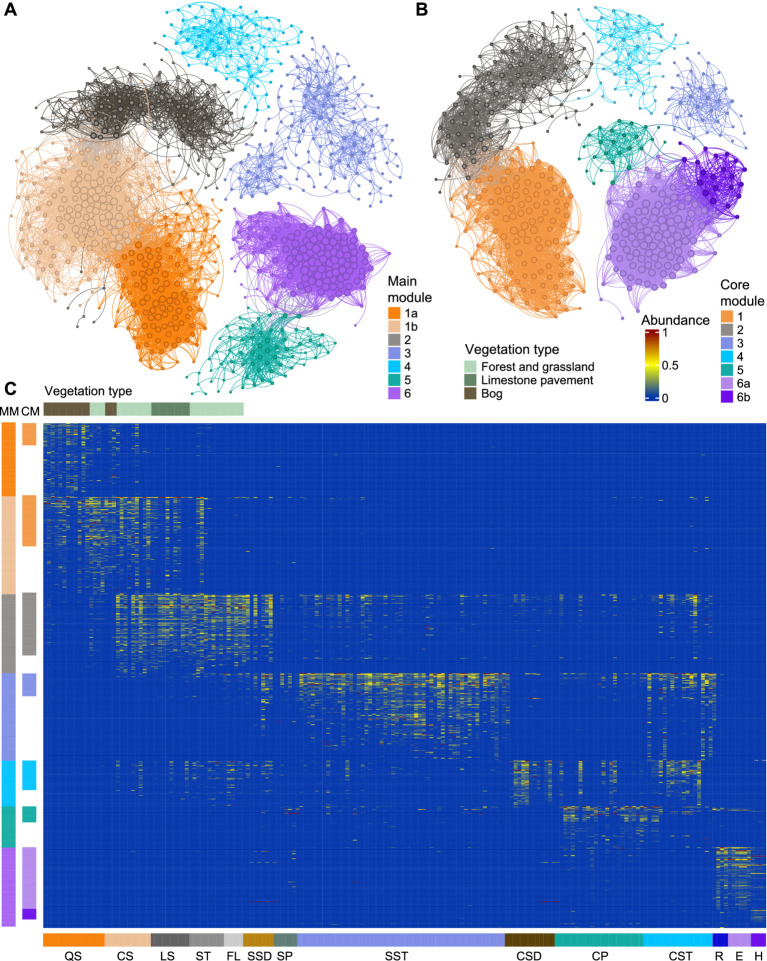
Association network showing co-occurrence of prokaryotes across the interconnected karst landscape: **(A)** Network analysis of the entire dataset **(B)** Network analysis of the core microbiome. Node sizes denote relative node abundance. **(C)** Heatmap showing the distribution of ASVs from the main co-occurrence modules of the entire dataset across different environmental types (min–max normalized relative abundance values, 0–1). MM, main modules from the entire dataset; CM, modules from the core network.

Co-occurrence network analysis of the core microbiome yielded a network comprising 468 ASVs connected by 7,951 significant edges, whereas analysis on the complete karst dataset yielded a network of 889 ASVs and 11,997 edges (Figures 5A,B). Modularity analysis identified six major modules in both networks. Full-network modules (MMs) represented expansions of their corresponding core modules (CMs) through the incorporation of additional ASVs with more restricted environmental distributions (Supplementary Table S7). Overall, node positions were largely conserved within all the modules in both the networks: 47.8% of ASVs retained hub status in both analyses. Only 1.9% of ASVs gained hub status exclusively in the full network, while 1.3% were hubs only in the core network. Additionally, a clear separation was observed between modules 1–2 and 5–6 in both the networks (Figures 5A,B), with MM1b and MM6 both showing high degree nodes (Supplementary Table S7). Several widely distributed core taxa, including *Rhodoferax*, SAR11 clade IIIb, and *Pseudomonas*, were not assigned to any co-occurrence module and were absent from both networks.

Module 1 (CM1/MM1a-b) was primarily distributed in QS and CS soils, with limited representation in LS soils. In the full network, it expanded and was subdivided into MM1a and MM1b (Figure 3C). MM1a comprised ASVs largely restricted to QS soils and was enriched in *Desulfobacterota* and *Pseudomonadota*, including *Methylocapsa* and *Methylocystis*, as well as spatially restricted taxa from the expansion, such as *Gallionella*, *Sideroxydans*, and the methanogenic archaeon *Methanoregula*. FAPROTAX annotations indicated both aerobic and anaerobic heterotrophy, including fermentation. Additional functions included nitrogen fixation, sulfate respiration, methanogenesis, as well as methylotrophy and methanotrophy (Supplementary Table S8). MM1b consisted of more broadly distributed soil ASVs shared across QS, CS, and LS soils and was dominated by *Acidobacteriota* [*Terriglobales* and Subgroup-2 (SILVA, release 138.1)], *Pseudomonadota*, and *Planctomycetota*. FAPROTAX annotations predominantly indicated aerobic heterotrophy. Additional functions included hydrogen oxidation, with minor contributions from fermentation, nitrate reduction, and nitrogen fixation (Supplementary Table S8). Module 2 (CM2/MM2) consisted of two tightly connected subclusters present in both networks: one restricted to soils and the other additionally spanning sediments, cave pools, and cave and surface streams. Expansion in MM2 only incorporated 26 additional ASVs primarily with terrestrial distributions and included *Acidobacteriota*, *Pseudomonadota*, *Planctomycetota*, and *Verrucomicrobiota*. FAPROTAX indicated dominance of aerobic heterotrophy, with the presence of anaerobic chemoheterotrophy. Nitrogen cycling processes were also prominent in this module, with a strong contribution from nitrification (ammonia oxidation) (Supplementary Table S8).

Module 3 (CM3/MM3) was distributed mainly across surface streams and pools and, to a lesser extent, subsurface streams and sediments, and showed the lowest average node degree (6.06) and betweenness centrality (206.13) (Supplementary Table S7). CM3 included unclassified *Rhizobiales* and *Rhodoferax* (*Pseudomonadota*) as hubs. MM3 showed the largest expansion among all modules, recruiting 105 additional ASVs spanning rivers, surface streams, cave streams, and surface sediments, while remaining absent from soils. It was mainly driven by additional *Pseudomonadota*, *Bacteroidota*, *Cyanobacteriota*, and nitrogen-cycling taxa such as *Nitrospira* and *Nitrosomonadaceae*. FAPROTAX-based functional assignment showed dominance of aerobic heterotrophy and phototrophy. Additional functions included nitrate reduction, nitrogen fixation, ureolysis, and fermentation (Supplementary Table S8).

Module 4 (CM4/MM4) was associated primarily with subsurface environments, particularly cave streams and sediments, with limited representation in ST and FL soils. Nodes in CM4 were typically characterized by low degree but moderate to high betweenness centrality. The core module included *Nitrosomonadaceae*, *Nitrospiraceae*, *Nitrosopumilales*, *Ferrovibrionales*, *Gallionellaceae*, *Anaerolineales*, and *Methylomirabilota*. The expanded module also incorporated *Gammaproteobacteria* PLTA-13 and *Burkholderiales* TRA3-20 (SILVA, release 138.1). FAPROTAX indicated a combination of aerobic and anaerobic heterotrophy. Among all modules, this one showed the strongest signal for nitrification (ammonia oxidation), with additional contributions from iron and hydrogen oxidation (Supplementary Table S8).

Module 5 (CM5/MM5) was primarily linked to subsurface pools and streams, with key ASVs including *Methylotenera*, *Methylobacter*, unclassified *Methylomonadaceae*, *Sediminibacterium*, *Limnohabitans*, and *Flavobacteriaceae*. Expansion in MM5 occurred through the inclusion of additional ASVs affiliated with *Verrucomicrobiota* and *Gammaproteobacteria*, i.e., *Crenothrix*. FAPROTAX annotations indicated both aerobic and anaerobic heterotrophy, together with significant contributions from methylotrophy and methanotrophy (Supplementary Table S8).

Module 6 (CM6a-b/MM6) encompassed lake-associated communities, where the core network consisted of two submodules, with CM6b restricted to the lake hypolimnion. Module six showed the smallest expansion, with MM6 incorporating only eight additional ASVs, which nevertheless increased connectivity between CM6a and CM6b. Highly connected taxa in this module included members of the CL500-29 marine group (*Planctomycetota*), *Sediminibacterium* (*Bacteroidota*), *Pedosphaeraceae* SH3-11 (SILVA, release 138.1, *Verrucomicrobiota*), and *Methylopumilus* (*Pseudomonadota*). FAPROTAX annotations revealed aerobic heterotrophy and phototrophy (cyanobacteria), with a distinct contribution from methylotrophic taxa, as well as nitrogen cycling (Supplementary Table S8).

Notably, ASVs contributing to the closer taxonomic similarity between cave pools and Lake Thun strata (Figure 2) were almost absent within the detected modules (only few within MM5 and MM6). Shared taxa between cave pools and the hypolimnion included members of *Gammaproteobacteria*, namely families *Nitrosomonadaceae*, *Methylophilaceae*, *Methylomonadaceae*, as well as genera *Limnohabitans* and *Rhodoferax*, and ASVs affiliated with *Nanopelagicales* (*Actinomycetota*), *Verrucomicrobiota*, and *Bacteroidota*. Compositional similarity between cave pools and the epilimnion was associated with an additional contribution of *Alcaligenaceae* (*Gammaproteobacteria*) and *Alphaproteobacteria*.

## Discussion

Our study provides a comprehensive view of bacterial community composition across an interconnected karst landscape spanning 14 distinct environment types and steps beyond the scope of cross-environmental studies that are usually restricted to spatially adjacent sites (Griffin et al., 2017; Hermans et al., 2020; Zhu et al., 2021). This allows us to distinguish bacterial communities shaped by local physicochemical conditions from those influenced by hydrological transfer and connectivity within aquatic networks.

Alpha-diversity trends observed in our study confirm previous reports, with terrestrial environments showing higher diversity than aquatic systems (Chau et al., 2011; Yang et al., 2025). Within terrestrial habitats, the most distinct environment identified by NMDS and RDA analyses, QS soils, also displayed the lowest average diversity and richness among soil types. A similar pattern was detected within aquatic systems, where lake strata showed the lowest average alpha diversity. In contrast, soil environments with higher connectivity, as well as aquatic environments positioned at the top of the hydrological network and associated with shorter water retention times, exhibited higher diversity indices. Thus, both species selection and dispersal enhancement shape diversity within the studied landscape. A comparable decrease in diversity, coinciding with increasing water retention time, has previously been reported within hydrological networks (Ruiz-González et al., 2015; Savio et al., 2015). Notably, alpha-diversity did not differ between the surface and subsurface components of the hydrological network.

Modules detected within co-occurrence networks revealed clear environmental signatures and distinct metabolic strategies but also indicated the transfer of associated community members across the landscape. Comparable modular patterns have been described in both soil and aquatic microbial networks, where co-occurring taxa often exhibit complementary metabolic roles beside co-variation in abundance (Faust and Raes, 2012; Banerjee et al., 2018; Mukherjee et al., 2024).

In the soil-associated modules identified here, MM1a (distributed within QS soils) showed a clear association with peat bog vegetation. This was indicated by the prevalence of taxa adapted to anaerobic or microaerobic metabolisms, including sulphate-reducing *Desulfobacterota*, methanogenic archaea, aerobic methanotrophs, and microaerophilic iron oxidizers (Pester et al., 2010; Bodelier and Dedysh, 2013; Tan et al., 2019). Bog environments appear to promote syntrophic interactions among taxa with highly specific metabolic capabilities, which together can cope with heterogeneous redox conditions and patchy substrate availability (e.g., sulphate and metals).

In contrast, the MM1b module lacked any associations with peat bogs or anoxic conditions and mainly included taxa commonly reported from mineral soils structured according to pH and substrate quality (Lauber et al., 2009; Rousk et al., 2010; Dedysh and Ivanova, 2019). This module was also more widely distributed across the environments and showed pronounced lineage turnover within *Acidobacteriota* along pH gradients, in line with previous reports (Sait et al., 2006; Kielak et al., 2016). The dichotomy between MM1a and b shows how hydrology-influenced vegetation effects can reorganize microbial interactions even in soils originated from the same parent rock.

MM2 appeared strongly expanded across environments, highlighting a soil-derived community that seemed to partially persist following hydrological export into sediments and aquatic environments. The MM2 module mainly brought together representatives of the *Burkholderiaceae*, *Nitrosomonadaceae*, *Sutterellaceae*, *Sphingomonadaceae*, and *Chitinophagaceae* families. Members of these taxa are well known for their metabolic flexibility and high tolerance to disturbances, and ability to adapt well to fluctuating resources and physicochemical conditions (Fierer et al., 2007; Newton et al., 2011; Crump et al., 2012; Gounand et al., 2018). This pattern suggests that MM2 members detected in aquatic environments and sediments may originate from hydrologically driven community coalescence, a process known to favor taxa with high physiological tolerance in terrestrial-aquatic transition zones (Rillig et al., 2016; Mansour et al., 2018). However, the absence of this module from aquatic systems with longer water retention times suggests its reduced capacity for long-term establishment.

Modules 3–6 were primarily associated with aquatic environments. MM3 was defined by surface and subsurface streams, which are characterized by short water retention times, frequent disturbance, and recurrent inputs of organic matter. The main representatives of this module were affiliated with *Bacteroidota*, particularly the orders *Flavobacteriales* and *Cytophagales*, together with diverse *Cyanobacteriota* and members of the *Burkholderiaceae* family. These taxa are core members of freshwater stream microbiomes, where they sustain by a balance between local growth and continuous replacement driven by hydrological mass effects (Besemer et al., 2012; Ruiz-González et al., 2015; Shabarova et al., 2021). Despite the presence of closely related, disturbance-tolerant taxa in both MM2 and MM3, the two modules showed markedly different distributions across environments, reflecting a separation between soil-derived and freshwater-associated communities.

The MM4 module was associated primarily with subsurface aquatic habitats and additionally detected in a subset of soil samples and surface streams. The taxonomic composition of this module pointed toward metabolisms characteristic of redox interfaces and microoxic conditions. It consisted of nitrifying and iron-oxidizing prokaryotes (i.a., *Nitrosomonadaceae*, *Nitrospiraceae*, *Nitrosopumilales*, *Gallionellaceae*), and nitrite-dependent methane oxidizers *Methylomirabilota* (Ettwig et al., 2010; Laufer et al., 2016; Shen et al., 2020). Assemblages with similar taxonomic and functional composition have been described in groundwater, sediments, and hyporheic zones (Mosley et al., 2022; Shen et al., 2022; Bech et al., 2023; Vuillemin et al., 2024). Overall, MM4 appears to represent a redox-driven assemblage shaped by a specific physicochemical niche rather than by discrete environmental boundaries.

MM5 represented a largely cave-restricted module associated primarily with cave pools, although a few individual ASVs were also detected in streams, rivers, and lake samples. The central feature of the module was represented by C1 metabolism and brought together obligate methanotrophs affiliated with *Methylococcales* (e.g., *Crenothrix*, *Methyloglobulus*, *Methylobacter*), with methylotrophic taxa such as *Methylotenera* and members of the *Beijerinckiaceae* (Orata et al., 2018; Taubert et al., 2019; Marín and Arahal, 2014), and aerobic decomposers. Previous studies on a smaller subset of cave pools from this region have shown that under stagnant, oxygenated conditions only methylotrophic taxa persist over time, while methanotrophs are detectable only during flooding events and fail to maintain populations for extended periods thereafter (Shabarova and Pernthaler, 2010; Shabarova et al., 2013, 2014). Still, it remains uncertain whether such dynamics apply uniformly to all cave pools studied here. Localized methane availability remains a possibility, as cave pools could receive methane via groundwater inflow, sedimentary anoxic niches, or via diffusion from more profound karst conduits, despite overall oxygenated conditions (Webster et al., 2018; Fernandez-Cortes et al., 2015). This module represented a distinct subsurface community, indicating an unexpected degree of compartmentalization within the hydrological network.

Finally, MM6 illustrated a classical freshwater plankton module defined by recurrent freshwater bacterial lineages such as *Planktophila* and other *Nanopelagicales* (hgcI clade, *Actinomycetota*), *Polynucleobacter*, *Limnohabitans*, and *Methylopumilus*, together with diverse *Bacteroidota* and *Planctomycetota* (Chiriac et al., 2025; Hahn and Hoetzinger, 2019; Kasalický et al., 2013; Salcher et al., 2019; Chiriac et al., 2023). This association of taxa is very similar to the types of microbial communities often reported in pelagic lake waters, where long water retention times, stable stratification, and sustained primary production favor the proliferation of specialized, slow-growing freshwater taxa with high substrate affinity and streamlined genomes (Newton et al., 2011; Salcher et al., 2015; Pernthaler, 2017; Chiriac et al., 2023). The fact that dominant members were represented by small-celled *Actinomycetota* (hgcI clade), together with oligotrophic *Alphaproteobacteria* and *Verrucomicrobiota* clearly indicates adaptations to low nutrient concentrations and the efficient utilization of dissolved organic matter, a hallmark of lake plankton communities. *Bacteroidota* likely contribute to the processing of polymeric organic matter resulting from phytoplankton, while *Cyanobacteriota* such as *Cyanobium* indicate coupling to primary production (Xie et al., 2024).

## Conclusion

This study provides a unique system-level perspective on microbial community assembly across an interconnected karst landscape. By integrating hydrologically linked terrestrial, surface, and subsurface environments, we revealed how multiple factors interact to drive community assembly across environmental boundaries. Lake samples represented the most isolated assemblages, likely indicating underlying long-term selection processes promoted by extended water retention times. Distinct physicochemical conditions and hydrologically structured vegetation led to the establishment of heterogeneous yet metabolically well-defined communities in terrestrial environments. Less specialized and presumably more stress-tolerant soil fractions showed evidence of coalescence into streams and sediments, where hydrological forcing sustained the dominance of copiotrophic taxa. While surface and subsurface streams shared both of these features, microbial communities in cave pools displayed a distinct subsurface signature centered on C1-compound metabolism and showed the closest compositional similarity to those found in lakes. Additionally, we identified a cluster of taxa associated with nitrification and metal-oxidation processes which was crossing several environmental boundaries. Altogether, we showed how hydrology and physicochemical gradients structure microbial communities across environments and provided an integrative overview of the selective forces driving both compartmentalization and connectivity within a karst landscape.

## Data Availability

The datasets generated for this study have been deposited in the European Nucleotide Archive (ENA) and the National Centre for Biotechnology Information (NCBI) under BioProject accession number PRJEB106094 (https://www.ebi.ac.uk/ena/browser/view/PRJEB106094; https://www.ncbi.nlm.nih.gov/bioproject/?term=PRJEB106094).
