# Supplementary material for: From soils to lake: interplay between hydrology and local environmental settings drives species selection across a karst landscape
Source: Front Microbiol. 2026 May 13;17:1813326. doi: 10.3389/fmicb.2026.1813326 (PMC13212123; doi:10.3389/fmicb.2026.1813326)
Supplement: Supplementary file 1 [file Data_Sheet_1.DOCX]

From soils to lake: Interplay between hydrology and local environmental settings drives species selection across a karst landscape

Anusha Priya Singh ^1,2^, Paul-Adrian Bulzu^1^, Vojtech Lanta^3^, Pavel Chaloupský^4^, Michaela M. Salcher^1^, Tanja Shabarova^1^

1. Department of Aquatic Microbial Ecology, Institute of Hydrobiology, Biology Centre CAS, Ceske Budejovice, Czechia
2. Faculty of Science, University of South Bohemia, Ceske Budejovice, Czechia
3. Institute of Botany CAS, Pruhonice, Czechia.
4. Department of Chemistry and Biochemistry, Faculty of AgriSciences, Mendel University in Brno, Brno, Czechia

Supplementary text and figures

# Supplementary text

## Site description

## The study region accounted for the diverse mosaics of habitat types, characterized by distinct vegetation formations.

## (1) Limestone pavements formed a predominantly rocky environment with patches of shallow and soil layer. The vegetation consisted of scattered individuals of woody plants (*Pinus sylvestris, Rhododendron hirsutum, Juniperus communis*), ferns (*Lastraea limbosperma, Athyrium filix-femina*), grasses (*Agrostis capillaris, Poa alpina*) and the heather *Calluna vulgaris*.

## (2) Peatbogs were dominated by *Sphagnum* spp. and other bryophytes (e.g., *Polytrichum commune, P. juniperinum*), accompanied by sedges (*Carex limosa, C. nigra*), low-growing shrubs (*Vaccinium uliginosum, Empetrum nigrum*) and cottongrasses (*Eriophorum vaginatum, E. angustifolium*).

## (3) Alpine meadows were species-rich habitats supporting a variety of grasses (*Deschampsia cespitosa, Holcus mollis, Festuca rubra*), light-demanding herbs (*Arnica montana, Silene vulgaris, Bellidiastrum michelii*) and patches of dwarf shrubs (*Vaccinium myrtillus, V. vitis-idaea*).

## (4) Closed-canopy forests were dominated by Norway spruce (*Picea abies*), with admixtures of broadleaf tree species (*Fagus sylvatica, Acer pseudoplatanus, Sorbus aucuparia*) and other conifers (*Abies alba*) at lower elevations. The understory was rather sparse, mainly composed of grasses and sedges (*Calamagrostis villosa, Brachypodium sylvaticum, Carex sylvatica, Luzula sylvatica*), shade-tolerant herbs (*Petasites albus, Oxalis acetosella, Campanula persicifolia, Helleborus niger*) and bryophytes (*Brachythecium* sp., *Leucobryum glaucum, Hypnum cupressiforme, Rhizomnium* sp.).

## Description of the water chemistry variables

## The aquatic systems in this landscape exhibited distinct physico-chemical characteristics and bacterial abundances varied markedly across the hydrological network. pH values ranged from neutral to slightly alkaline (7.24-8.46), likely influenced by limestone dissolution (Fig S1.a); however, surface pools located in the areas Wagemoos and Seefeld (Supplementary Fig. S1) were notably acidic. Temperature fluctuated considerably in surface systems, while the subsurface network maintained relatively stable thermal conditions, and thermal stratification was observed within the lake. Conductivity mirrored alkalinity patterns, with higher values recorded in streams than pools and consistent readings across lake depths. RP and RRP levels were higher in the subsurface, while DOC concentrations were greater in pools than streams. Both DN and NO₃⁻ were elevated in cave environments. Cl⁻ concentrations were highest in cave streams, SO₄²⁻ in surface systems and F⁻ reached maximum concentrations in rivers. Na⁺ and K⁺ had highest concentrations in surface pools and streams, Ca²⁺ levels were highest in all lotic systems, while Mg²⁺ was elevated only in surface systems. Ion concentrations were generally higher in lake hypolimnion than epilimnion. Data from previous sampling campaigns revealed seasonal variation, particularly in the lake hypolimnion, which showed increased concentrations of DN, NO₃⁻, K⁺, F⁻ during the main sampling campaign. Bacterial abundance was highest in pools, moderate in lake epilimnion and rivers and lowest in streams and lake hypolimnion. Full chemistry data is shown in Supplementary Table S1.

## RDA results

**Soil environments**

Method: RDA with supplementary variables. Partial variation is 10021.084, explanatory variables account for 42.76% (Adjusted explained variation is 37.04%).

Summary table:

| *Statistic* | *Axis 1* | *Axis 2* | *Axis 3* | *Axis 4* |
| --- | --- | --- | --- | --- |
| *Eigenvalues* | *0.2432* | *0.1026* | *0.0130* | *0.0028* |
| *Explained variation (cumulative)* | *28.75* | *40.89* | *42.43* | *42.76* |
| *Pseudo-canonical correlation* | *0.7560* | *0.6747* | *0.6420* | *0.4021* |
| *Explained fitted variation (cumulative)* | *67.25* | *95.62* | *99.23* | *100.00* |
| *Pseudo-canonical correlation* | *0.9613* | *0.4350* | *0.6251* | *0.1417* |

Forward selection results:

| - *Name* | - *Explains %* | - *Contribution %* | - *pseudo-F* | - *P* |
| --- | --- | --- | --- | --- |
| - *pH* | - 26.3 | - 59.3 | - 15.3 | - 0.002 |
| - *Dry weight* | - 5.6 | - 12.6 | - 3.4 | - 0.024 |
| - *PB (peatbogs)* | - 7.3 | - 16.4 | - 4.9 | - 0.002 |
| - *Stream present* | - 3.6 | - 8.2 | - 2.5 | - 0.052 |
| - *Stream absent* | - 3.6 | - 8.2 | - 2.5 | - 0.058 |

**Aquatic environments**

Method: RDA with supplementary variables
Total variation is 475.60481, explanatory variables account for 32.11% (Adjusted explained variation is 25.85%)

Summary table:

| *Statistic* | - *Axis 1* | - *Axis 2* | - *Axis 3* | - *Axis 4* |
| --- | --- | --- | --- | --- |
| - *Eigenvalues* | - 0.1825 | - 0.0799 | - 0.0233 | - 0.0162 |
| - *Explained variation (cumulative)* | - 18.25 | - 26.24 | - 28.57 | - 30.19 |
| - *Pseudo-canonical correlation* | - 0.7303 | - 0.5814 | - 0.4752 | - 0.5161 |
| - *Explained fitted variation (cumulative)* | - 56.85 | - 81.73 | - 88.99 | - 94.03 |
| - *Pseudo-canonical correlation* | - 0.7527 | - 0.6567 | - 0.4554 | - 0.4184 |

Forward selection results:

| *Name* | - *Explains %* | - *Contribution %* | - *pseudo-F* | - *P* |
| --- | --- | --- | --- | --- |
| *Alkalinity* | - 8.4 | - 19.4 | - 7.5 | - 0.002 |
| *Temperature* | - 6.2 | - 14.3 | - 6.1 | - 0.002 |
| *F^-^* | - 4.6 | - 10.7 | - 5.1 | - 0.002 |
| *PR* | - 4.1 | - 9.4 | - 4.2 | - 0.002 |
| *DOC* | - 3.9 | - 9.0 | - 3.6 | - 0.0014 |
| *Mg^2+^* | - 2.5 | - 5.9 | - 2.8 | - 0.018 |
| *NO_3_^-^* | - 2.4 | - 5.5 | - 2.4 | - 0.042 |

#
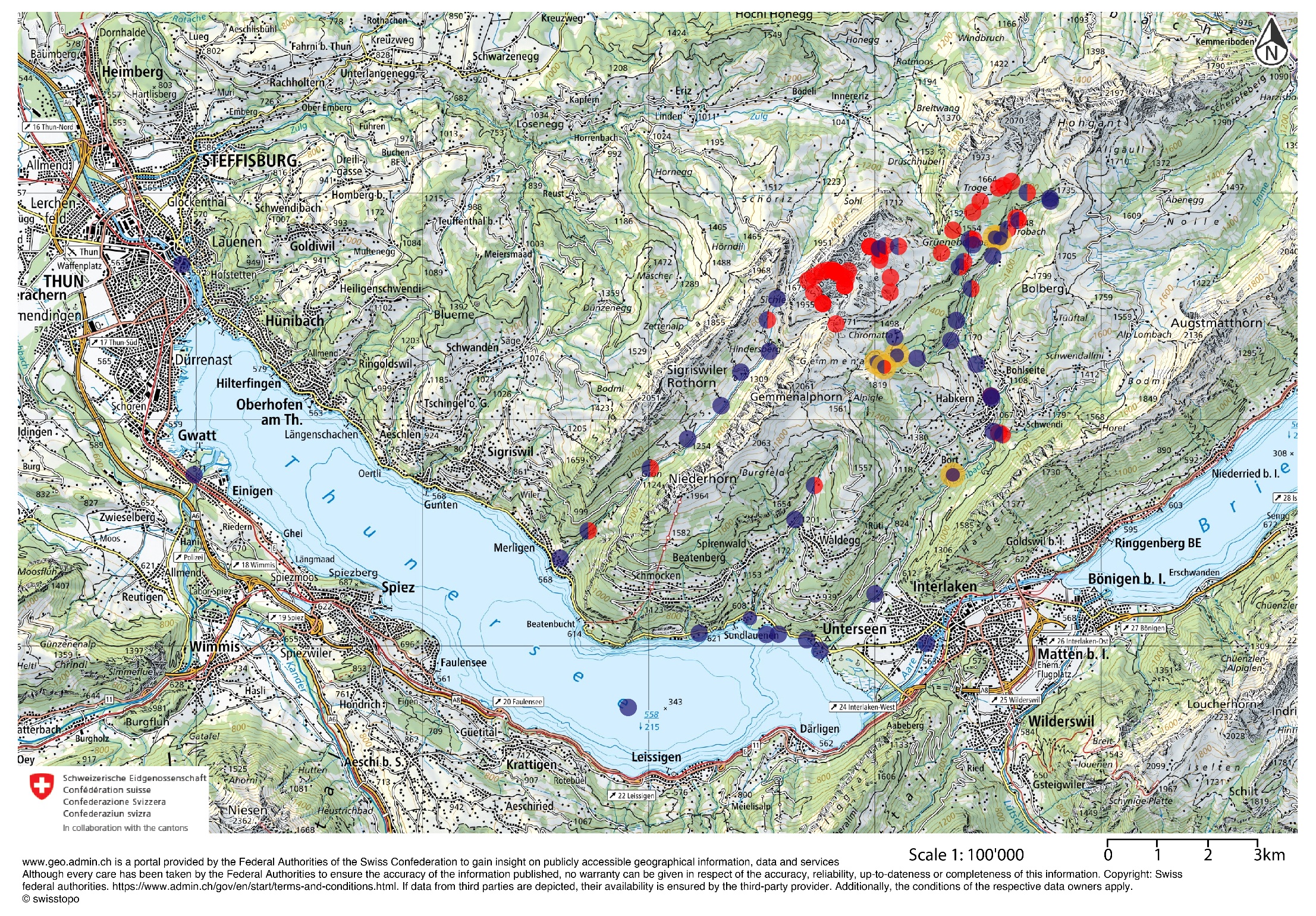
Supplementary figures

**Supplementary Figure S1.** Study site located on the northern shore of Lake Thun, Switzerland. Blue circles indicate water sampling sites, red circles indicate soil sampling sites, and collocated soil and water sampling sites are shown as half circles. Sediment sampling sites are indicated by yellow outer circles. Cave sampling sites are not shown. The map used for this figure was provided by the Federal Office of Topography (swisstopo), Switzerland.

**Supplementary Figure S2.** Alpha diversity of microbial communities across environments measured by A. Shannon index B. Observed richness. Boxplots show the distribution of values for each site. Different letters above the boxes indicate statistically significant differences among sites based on Dunn’s post-hoc tests following Kruskal–Wallis analyses (adjusted p < 0.001). Sites sharing at least one common letter are not significantly different from each other, whereas sites without a common letter differ significantly.

**Supplementary Figure S3.** Nonmetric multidimensional scaling (NMDS) plot based on Bray-Curtis dissimilarities between prokaryotic communities resolved at phylum level. Colours and shapes indicate environment type.
